# *Toxoplasma gondii*, *Sarcocystis* sp. and *Neospora caninum-*like parasites in seals from northern and eastern Canada: potential risk to consumers

**DOI:** 10.1016/j.fawpar.2019.e00067

**Published:** 2019-11-02

**Authors:** Sarah J. Reiling, Lena Measures, Sandy Feng, Ryan Boone, Harriet Merks, Brent R. Dixon

**Affiliations:** a Bureau of Microbial Hazards, Food Directorate, Health Canada, Ottawa, ON, K1A 0K9, Canada; b Fisheries and Oceans Canada, Maurice Lamontagne Institute, Mont-Joli, QC, G5H 3Z4, Canada

**Keywords:** *Toxoplasma*, *Sarcocystis*, *Neospora*, Seals, Zoonotic, Canada

## Abstract

Zoonotic parasites of seals that are harvested for food may pose a health risk when seal meat or organ tissues of infected animals are eaten raw or undercooked. In this study, 124 tissue samples from 81 seals, comprising four species, were collected from northern and eastern Canada. Tissues from 23 ringed seals (*Pusa hispida*), 8 hooded seals (*Cystophora cristata*), 21 harp seals (*Pagophilus groenlandicus*), and 29 grey seals (*Halichoerus grypus*) were tested for parasites of the Sarcocystidae family including *Toxoplasma gondii*, *Sarcocystis* spp., and *Neospora* spp. using nested PCR followed by Sanger sequencing. *Toxoplasma gondii* DNA was present in 26% of ringed seals, 63% of hooded seals, 57% of harp seals, and 31% of grey seals. *Sarcocystis* sp. DNA was found in 9% of ringed seals, 13% of hooded seals, 14% of harp seals, and 4% of grey seals, while *N. caninum-*like DNA was present in 26% of ringed seals. While it is unclear how pinnipeds may become infected with these protozoans, horizontal transmission is most likely. However, one harp seal pup (4 days old) was PCR-positive for *T. gondii*, suggesting vertical transmission may also occur. Phylogenetic analysis of the 18S gene region indicates that *Sarcocystis* sp. in these seals belongs to a unique genotype. Furthermore, this study represents a new host report for *T. gondii* in harp seals, a new host and geographic report for *N. caninum-*like parasites in ringed seals, and four new hosts and geographic reports for *Sarcocystis* sp. These results demonstrate that parasites of the Sarcocystidae family are prevalent in northern and eastern Canadian seals. While the zoonotic potential of *Sarcocystis* sp. and the *N. caninum*-like parasite are unclear, consumption of raw or undercooked seal meat or organ tissues pose a risk of *T. gondii* infection to consumers.

## Highlights

• Tissues from ringed, hooded, harp and grey seals in Canada were PCR-positive for *Toxoplasma*, *Sarcocystis* and *Neospora*.
• Raw or undercooked seal meat may pose a risk for zoonotic transmission of *T. gondii* to consumers.
• The risk for zoonotic transmission of *Sarcocystis* sp. and the *Neospora caninum*-like parasite is unknown.

## 1 Introduction

Seal meat and organs are important country foods of Inuit in Arctic and subarctic Canada and Greenland. In addition to subsistence harvests, some seal species are also harvested commercially in a government regulated sustainable harvest in eastern Canada (Hammill and Stenson, 2007; DFO, 2016), and seal meat is available at retail in this region. It is also offered in restaurants in metropolitan areas such as Toronto, Montreal and Quebec City, where the meat is often served as rare seal steak or as seal tartare. Seals are processed using specific guidelines for quality and food safety (Canadian Food Inspection Agency, 2014; Daoust and Stacey, 2014).

Seven pinniped species are hunted in Canada, including: walrus (*Odobenus rosmarus*), bearded seals (*Erignathus barbutus*), ringed seals (*Pusa hispida*), harbour seals (*Phoca vitulina*), hooded seals (*Cystophora cristata*), harp seals (*Pagophilus groenlandicus*), and grey seals (*Halichoerus grypus*). Most of these species are hunted for subsistence purposes by Inuit and others, but the latter three species are commercially hunted, with meat and by-products being sold to restaurants and exported. These pinnipeds have different distributions, abundance and, for some species, seasonal migrations which affect their availability to hunters. They also have different diets which affect their exposure to parasites and thus pose differential zoonotic risks to human consumers.

Ringed seals have a northern circumpolar distribution, are widespread in Arctic and subarctic Canada, and an estimated 100,000 are harvested annually for subsistence (Kingsley and Byers, 1998). Harp seals are separated into three populations based on specific pupping sites: Northwest Atlantic, Greenland Sea near Jan Mayen (West Ice) and White Sea/Barents Sea (East Ice). The abundant Northwest Atlantic harp seal population (DFO, 2012) is commercially harvested in Atlantic Canada from 307,000 (in 1956) to 40,000 (in 2011) annually (Stenson, 2014). Hooded seals are separated into two breeding herds (same genetic population) based on specific pupping sites: Northwest Atlantic and Greenland Sea (West Ice) (Coltman et al., 2007). The Northwest Atlantic population has subsistence and commercial harvests in Atlantic Canada ranging from 5905 (in 1946) to 0 (in 2006), and less than 400 annually since 1999 (Hammill and Stenson, 2006). Since 1964, commercial harvest of hooded seals is not permitted in the Gulf of St. Lawrence, and the majority of the harvest occurs in Greenland (Stenson et al., 2006). Grey seals, distributed on both sides of the North Atlantic, are found in the Gulf of St. Lawrence and coastal Nova Scotia and Newfoundland. There is a small commercial hunt in the Magdalen Islands and eastern shore of Nova Scotia of less than 1700 animals in 2016 (DFO, 2017).

Little is known about the risk to humans from the consumption of seal meat containing zoonotic pathogens and parasites that they may carry. However, various studies have identified pathogens and parasites in marine mammals of concern to human health (Tryland, 2000; Jenkins et al., 2013; Daoust and Stacey, 2014; Tryland et al., 2014). In this study, we tested ringed seals, harp seals, hooded seals, and grey seals for the presence of the protozoan parasites *Toxoplasma gondii, Sarcocystis* spp., and *Neospora* spp.

*Toxoplasma gondii* is the most prevalent parasite infecting humans and other warm-blooded animals worldwide (Hill and Dubey, 2002). Approximately one to two billion people are infected with this protozoan parasite (Bahia-Oliveira et al., 2017). The definitive hosts of *T. gondii* are felids, which shed oocysts in their feces. Humans may become infected by accidental ingestion of oocysts in contaminated soil, water, or food. Another transmission route is the consumption of raw or undercooked meats or organs from intermediate hosts infected with *T. gondii* tissue cysts (Jones and Dubey, 2010). In most healthy adult humans, the infection is asymptomatic. When a woman is exposed to *T. gondii* for the first time during pregnancy, the parasite may be vertically transmitted to the fetus (Hill and Dubey, 2002), and may result in death or severe illness Immunocompromised patients may develop toxoplasmic encephalitis (Hill and Dubey, 2002). Some Inuit communities show a high level of exposure to *T. gondii,* with almost 60% seroprevalence in Nunavik, Quebec (Messier et al., 2009). This high seroprevalence was associated with handling or consuming country foods (Messier et al., 2009). Most animal species that are harvested in the Canadian North as country foods, including various terrestrial and marine mammals, birds, and fish, have tested positive for *T. gondii* (see Reiling and Dixon, 2019). Traditionally, some country foods are eaten raw, which increases the chance of contracting toxoplasmosis.

*Sarcocystis* spp. typically have prey-predator life cycles involving herbivores and carnivores as intermediate and definitive hosts, respectively. These parasites primarily infect skeletal muscle, heart muscle, and lymph nodes of the intermediate host (Fayer, 2004). Humans can serve as definitive hosts for *S. hominis* and *S. suihominis*, which are acquired from eating undercooked beef and pork, respectively (Fayer et al., 2015). Humans can also serve as intermediate hosts for other *Sarcocystis* spp., likely acquired by ingesting sporocysts from contaminated food or water, or in the environment (Fayer et al., 2015). Infection in humans causes the disease sarcocystosis, which is generally asymptomatic. In Southeast Asia, muscular sarcocystosis in humans was found to be 21% (Wong and Pathmanathan, 1992). To our knowledge, no *Sarcocystis* infections in humans have been documented in Canada except for travel-related cases to Southeast Asia (Esposito et al., 2014).

*Neospora caninum* is closely related to *T. gondii*, and earlier reports confused the two species (Dubey et al., 2002a). Dogs and other canids are the definitive host of *N. caninum* and oocysts are shed in the canid's feces (Donahoe et al., 2015). *Neospora caninum* may cause severe neuromuscular disease in dogs, resulting in paraparesis of their hind limbs (Dubey et al., 2007). In cattle, which serve as intermediate hosts, *N. caninum* may cause encephalitis and abortions and can be transmitted vertically (Moré et al., 2009). Antibodies to *N. caninum* were reported in 7% of human serum samples in the USA (Tranas et al., 1999), and in 6% of healthy adults (Lobato et al., 2006). *Neospora caninum* seroprevalence is significantly higher in HIV-infected patients (38%) and in patients with neurological disorders (18%) (Lobato et al., 2006). However, this parasite has not been detected in human tissues, thus its zoonotic potential has not been clearly demonstrated (Dubey et al., 2007).

The objective of this study was to determine the prevalence of *Toxoplasma*, *Sarcocystis*, and *Neospora* infections in four species of seals that are harvested for food in northern and eastern Canada. Results from this study will aid in evaluating the risk of transmission of these parasites to humans through the consumption of seal meat or organ tissues.

## 2 Material and methods

### 2.1 Samples

Ringed seals, *P. hispida*, (n = 19), 12 young-of-the-year (YOY) or juvenile females (10 Age = 0 (YOY), 1 Age = 2, 1 Age = 4) and 7 YOY or juvenile males (6 Age = 0, 1 Age = 1), were collected by Inuit hunters and sampled in 1993 and 1994 at Salluit, Nunavik, Quebec (62°13′N, 75°39′W) (Table S1). In addition, tissues from four ringed seals were collected from Inukjuak, Nunavik, Quebec (58°26′N 78°06′W) but no information was available on the age or sex of these animals. Harp seals, *P. groenlandicus,* (n = 21), 19 adult females, one YOY male and one YOY female, and hooded seals, *C. cristata*, (n = 8), adult females only, were shot under scientific permit issued by Fisheries and Oceans Canada and sampled in 2005 from breeding ice floes located west of the Magdalen Islands (47°23′N, 61°52′W) in the Gulf of St. Lawrence, Québec. Grey seals, *H. grypus,* (n = 29), 14 adult females and 15 adult males, were shot under scientific permit and sampled in 2012 from breeding colonies on Saddle Island (45°48′N 63°15′W) and Pictou Island (45°49′N 62°33′W), Nova Scotia.

Canine teeth were extracted from lower jaws for age determination of ringed and grey seals only. Thin cross-sections of teeth were made and the number of dentinal annuli were counted with one growth layer group = one year of age. Hooded and grey seals were aged based on total length and sexual maturity (only adults are present on the breeding ice floes). Seals were classified as YOY, juvenile or adult as described in Measures et al. (2004). The sex was determined in 77 of 81 seals (Table S1); 54 (70%) were female and 23 (30%) were male.

A total of 124 tissue samples were collected from 81 seals and included diaphragm (n = 53), brain (n = 28), heart muscle (n = 20), lung (n = 19) and skeletal muscle (n = 4). Tissue samples were stored at −20 °C.

### 2.2 DNA extraction

Tissue samples were thawed, and 1 g subsample of each was divided into two 500 mg aliquots which were used for DNA extraction. The cell lysis protocol was adapted from Opsteegh et al. (2010). To each aliquot, 625 μl of cell lysis buffer containing 100 mM Tris-HCl pH 8.0, 50 mM EDTA pH 8.0, 100 mM NaCl, 1% SDS, 2% 2-mercaptoethanol, and 5 mg/ml proteinase K (Sigma-Aldrich, Oakville, ON, Canada), and 100 μl of 0.1 mm glass beads and 0.7 mm zirconia beads (BioSpec Products, Burlington, ON, Canada) were added. Samples were homogenized 6500 rpm for 3 × 20 s using the Precellys 24 homogenizer (Bertin Technologies, Rockville, MD, USA) before incubating overnight at 45 °C. Aliquots were pooled into 15 ml conical tubes and 1.25 ml cell lysis buffer was added and incubated for 2 h at 45 °C.

To each homogenized tissue sample, 625 μl of 5 M NaCl and 510 μl of cetyl trimethylammonium bromide (CTAB)/NaCl (10% CTAB in 0.7 M NaCl) were added. Samples were incubated at 65 °C for 15 min. An equal volume of phenol/chloroform/isoamyl-alcohol (25:24:1) was added to the sample, followed by a 1.5 h incubation at room temperature (RT) while mixing on a Revolver™ Rotator (Labnet International, Edison, NJ, USA). The solution was then centrifuged at 3000×*g* for 15 min at 12 °C. The supernatant was dispensed into a new 15 ml conical tube. An equal volume of chloroform/isoamyl-alcohol (24:1) was added, and the samples were placed on a revolver for 1 h at RT. Samples were then centrifuged as indicated above. The supernatant was collected and 2 vol of cold 100% ethanol were added to precipitate the DNA. Samples were stored overnight at 4 °C for complete precipitation.

The precipitated DNA was pelleted at 3000×*g* for 20 min at 4 °C. An equal volume of 70% ethanol was added to wash the DNA pellet before centrifugation at 1000×*g* for 10 min at 4 °C. This step was repeated twice. The pellet was then transferred to a 1.5 ml LoBind tube (Corning Inc., Corning, NY, USA) and air dried until translucent. The dry pellet was resuspended in 150 μl of EB Elution Buffer (Qiagen, Mississauga, ON, Canada) at 50 °C for 4 h. The extracted DNA was stored at −20 °C.

### 2.3 Nested PCR

The gene regions, primers, and their respective nucleotide sequences that were used in this study are listed in Table 1. All tissues available for testing in this study were tested with B1 and 18S primers. *Sarcocystis*-specific primers were used to confirm *Sarcocystis* sp. All PCR reactions were performed with a total reaction volume of 25 μl containing 1 × concentration of a 5 × Green GoTaq Reaction Buffer, 2 mM of MgCl_2_, 200 μM of dNTPs, 0.625 U GoTaq Polymerase (all from Promega, Madison, WI, USA), 300 nM of each primer (Sigma-Aldrich Canada, Oakville, ON, Canada), 1 μl of template DNA, and UltraPure water (Invitrogen, Carlsbad, CA. USA). The DNA concentration, quantified using Nanodrop (ThermoFisher Scientific, Waltham, MA, USA), was normalized to 500 ng per reaction. PCR reaction was performed using the Mastercycler Nexus X2 thermocycler (Eppendorf, Hamburg, Germany) for all samples. Cycling conditions for all samples were: 95 °C for 2 min, 35 cycles of 94 °C for 30 s, 50–68 °C for 30 s, and 72 °C for 60 s, following by a final extension at 72 °C for 10 min, and final hold temperature of 10 °C. The annealing temperatures varied between primers and are listed in Table 1. Negative controls were added to each PCR run. Positive controls consisted of DNA extracted from *T. gondii* oocysts kindly donated by Dr. J. P. Dubey, USDA. While positive controls were not available for *Sarcocystis* sp. or *Neospora caninum*, *T. gondii* positive control was used as a negative control for these parasites.

**Table 1:** Primer sequences of the genes used for polymerase chain reactions.

Gene		Annealing Temp.	Primer	Primer sequence (5′-3′)	Reference
**18S (Sarcocystidae)**	outer PCR	68 °C	N-DIAGF2	CAATTGGAGGGCAAGTCTGGTGCCAGC	Nichols et al. (2003)
			N-DIAGR2	CCTTCCTATGTCTGGACCTGGTGAGT	Nichols et al. (2003)
	nested PCR	59 °C	CPB-DIAGF	AAGCTCGTAGTTGGATTTCTG	Nichols et al. (2003)
			SJR Toxo2R	GTGCAGGAGAAGTCAAGCATGACG	Present study
**18S (*Sarcocystis*)**	outer PCR	50 °C	Sarc Back OutF	AGTAATGATTAATAGGGACAGTTG	Wassermann et al. (2017)
			Sarc Back OutR	GTGAATGATCCTTCCGCAGGTTCA	Wassermann et al. (2017)
	nested PCR	50 °C	Sarc Back NestF	GCATTCGTATTTAACTGTCAGAGG	Wassermann et al. (2017)
			Sarc Back NestR	CTACGGAAACCTTGTTACGACTTC	Wassermann et al. (2017)
**B1 (*Toxoplasma*)**	outer PCR	58 °C	B1outF	GGAACTGCATCCGTTCATGAG	Di Guardo et al. (2011)
			B1outR	TCTTTAAAGCGTTCGTGGTC	Di Guardo et al. (2011)
	nested PCR	58 °C	B1intF	TGCATAGGTTGCAGTCACTG	Di Guardo et al. (2011)
			B1intR	GGCGACCAATCTGCGAATACACC	Di Guardo et al. (2011)

Positive samples as determined by gel electrophoresis were purified using either the QIAquick PCR purification kit or the QIAquick Gel Extraction kit (Qiagen, Mississauga, ON, Canada) following manufacturer's instructions.

### 2.4 Sanger sequencing

The purified PCR products were prepared for, and subjected to, bi-directional, cycle sequencing using BigDye Terminator v3.1 Cycle Sequencing Kit (Applied Biosystems, Waltham, MA, USA) as recommended by the manufacturer. Amplified sequence products were purified using Wizard MagneSil green (Promega, Madison, WI, USA) sequencing reaction clean-up system, and capillary electrophoresis was performed on a 3500 Genetic Analyser (Applied Biosystems, Waltham, MA, USA). Sequences were assembled, edited and aligned using SeqScape v3 software (Applied Biosystems, Waltham, MA, USA). Resulting consensus sequences were aligned with representative GenBank 18S sequence data from *T. gondii*, *Sarcocystis* spp. and *Neospora* spp., and trimmed to identical lengths of 441bp using BioEdit (Hall, 1999). Sequences are available through GenBank accession numbers MH514961-MH514967 for the *Sarcocystis*-positive samples, and GenBank accession numbers MH595863-MH595890 for the *Toxoplasma*- or *Neospora*-positive samples.

### 2.5 Molecular phylogenetic analysis by Maximum Likelihood method

The evolutionary history was inferred using the Maximum Likelihood method based on the Kimura 2-parameter model (Kimura, 1980). The tree with the highest log likelihood was used. Initial tree(s) for the heuristic search were obtained automatically by applying Neighbor-Join and BioNJ algorithms to a matrix of pairwise distances estimated using the Maximum Composite Likelihood (MCL) approach, and then selecting the topology with superior log likelihood value. Trees were drawn to scale, with branch lengths measured in the number of substitutions per site. All positions with less than 95% site coverage were eliminated. Evolutionary analyses were conducted in MEGA6 (Tamura et al., 2013).

## 3 Results

### 3.1 *Toxoplasma gondii* and *Sarcocystis* sp. DNA was present in all seal species tested

Depending upon the seal species and collection method, different tissues were available for testing, including muscle, brain, heart, lung, and diaphragm (Supplementary Table S1). PCR was performed on all tissues using 18S and B1 primers as described in Table 1. 18S Sarcocystidae primers were used to detect all parasites of the Sarcocystidae family. All Sarcocystis sp. positive samples were confirmed using a second, Sarcocystis-specific, nested 18S primer. Toxoplasma gondii was detected using 18S and B1 primers, however, not all tissues were found to be positive for both primers (Supplementary Table S1).

*Toxoplasma gondii, Sarcocystis* sp. and *N. caninum-*like DNA was detected in 40%, 9% and 7% of seals, respectively (Table 2). Analyses revealed *T. gondii* DNA in 26% of ringed seals, 63% of hooded seals, 57% of harp seals, and 31% of grey seals. *Sarcocystis* sp. DNA was found in 9% of ringed seals, 13% of hooded seals, 14% of harp seals, and 4% of grey seals. *Neospora caninum-*like DNA was only found in ringed seals from Salluit (26%).

**Table 2:** Prevalence of *Toxoplasma gondii*, *Sarcocystis* sp. and *Neospora caninum*-like parasites in seals from northern and eastern Canada.

Species	Location^a^	No. animals tested	*Toxoplasma gondii*-positive (%)	*Sarcocystis* sp.-positive (%)	*Neospora caninum*-like-positive (%)
Ringed seals (*Pusa hispida*)	Inukjuak, QC	4	1 (25)	2 (50)	0 (0)
Ringed seals (*Pusa hispida*)	Salluit, QC	19	5 (26)	0 (0)	6 (32)
	ringed seal total	23	6 (26)	2 (9)	6 (26)
Hooded seals (*Cystophora cristata*)	Magdalen Islands, QC	8	5 (63)	1 (13)	0 (0)
Harp seals (*Pagophilus groenlandicus*)	Magdalen Islands, QC	21	12 (57)	3 (14)	0 (0)
Grey seals (*Halichoerus grypus*)	Saddle Island, NS	5	2 (40)	0 (0)	0 (0)
Grey seals (*Halichoerus grypus*)	Pictou Island, NS	24	7 (29)	1 (4)	0 (0)
	grey seal total	29	9 (31)	1 (4)	0 (0)
	total	81	32 (40)	7 (9)	6 (7)

a QC, Quebec; NS, Nova Scotia.

All *Sarcocystis* sp.-infected animals of known sex were female; however, 97% of the seals harvested from the Magdalen Islands were female and the sex of the seals from Inukjuak was unknown. All animals from the Salluit cohort were <5 years of age; of the 6 *N. caninum-*like positive animals, 4 (67%) were female and 2 (33%) were male, and 5 of the 6 were YOY and one was Age = 1. For *T. gondii*-infected animals, the sex was known for 31 of 32 animals, of which 19 (61%) were female and 12 (39%) were male, which included one 4-day-old male harp seal pup (Supplementary Table).

### 3.2 Parasite infections differed amongst regions

**Fig. 1:**
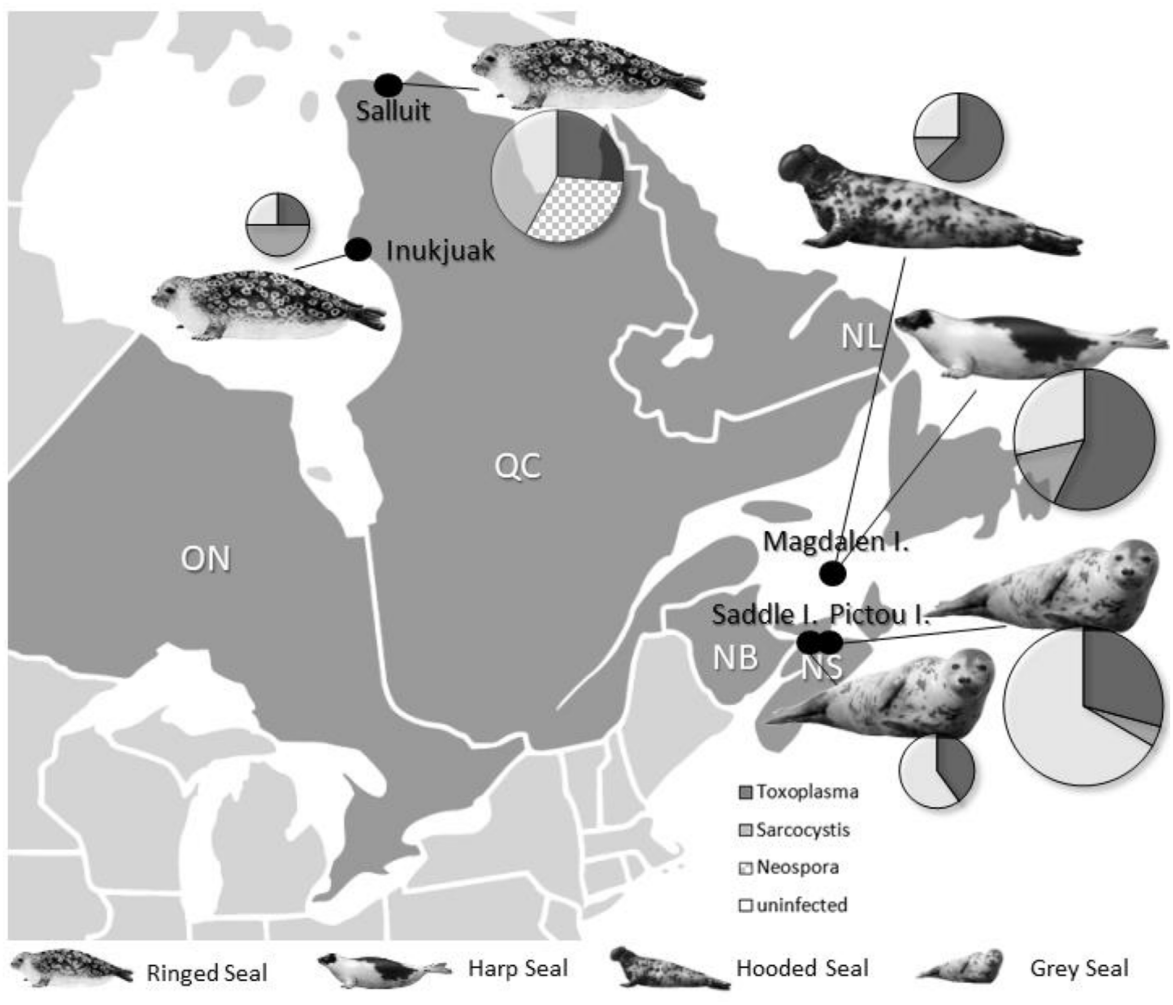
Harvesting locations of seal species: Salluit, Quebec (ringed seals, n = 19), Inukjuak, Quebec (ringed seals, n = 4), Magdalen Islands, Quebec (hooded seals, n = 8; harp seals, n = 21), Saddle Island, Nova Scotia (grey seals, n = 5), Pictou Island, Nova Scotia (grey seals, n = 24). The size of the pie charts represents the number of seals sampled per harvesting location. NB, New Brunswick; NL, Newfoundland and Labrador; NS, Nova Scotia; ON, Ontario; QC, Quebec.

The prevalence of *T. gondii*, *Sarcocystis* sp., and *N. caninum*-like parasites in the seal populations differed by harvest location (Fig. 1, Table 2, Table S1). *Toxoplasma gondii* DNA was detected in all seal species from all harvest locations (Fig. 1). *Sarcocystis* sp. DNA was detected in all seal species but only from three of five harvest locations, namely ringed seals from Inukjuak, grey seals from Pictou Island, and harp and hooded seals from the Magdalen Islands. As *Sarcocystis* sp. DNA was predominantly found in skeletal muscle (Table 3), and this tissue was not available from all seal species and harvest locations, some *Sarcocystis* infections may have been undetected. *Neospora caninum*-like DNA was detected in ringed seals (32%) from Salluit but not in ringed seals from Inukjuak, nor in any other seal species.

**Table 3:** Distribution of *Toxoplasma gondii*, *Sarcocystis* sp. and *Neospora caninum*-like parasites in seal tissues.

Tissue^a^	No. samples tested	*Toxoplasma gondii*-positive (%)	*Sarcocystis* sp.-positive (%)	*Neospora caninum*-like-positive (%)
Diaphragm	53	17 (32)	3 (6)	0 (0)
Brain	28	9 (32)	1 (4)	0 (0)
Heart muscle	20	6 (30)	1 (5)	0 (0)
Lung	19	5 (26)	0 (0)	6 (32)
Skeletal muscle	4	1 (25)	2 (50)	0 (0)
total	124	38 (31)	7 (6)	6 (5)

a Six individual seals were PCR-positive in more than one tissue.

### 3.3 *Toxoplasma gondii* was evenly distributed across all tissues

Tissues positive for *T. gondii* included diaphragm, brain, heart muscle, lung and skeletal muscle, in similar prevalences (Table S1, Table 3). *Sarcocystis* sp. appeared to have a preference for skeletal muscle compared to diaphragm, brain, heart muscle and lung. *Neospora caninum*-like DNA was detected only in lung tissues from ringed seals harvested in Salluit as it was the only tissue available from this particular seal cohort (Table S1).

### 3.4 *Neospora caninum*-like parasites are closely related to *Toxoplasma gondii*

**Fig. 2:**
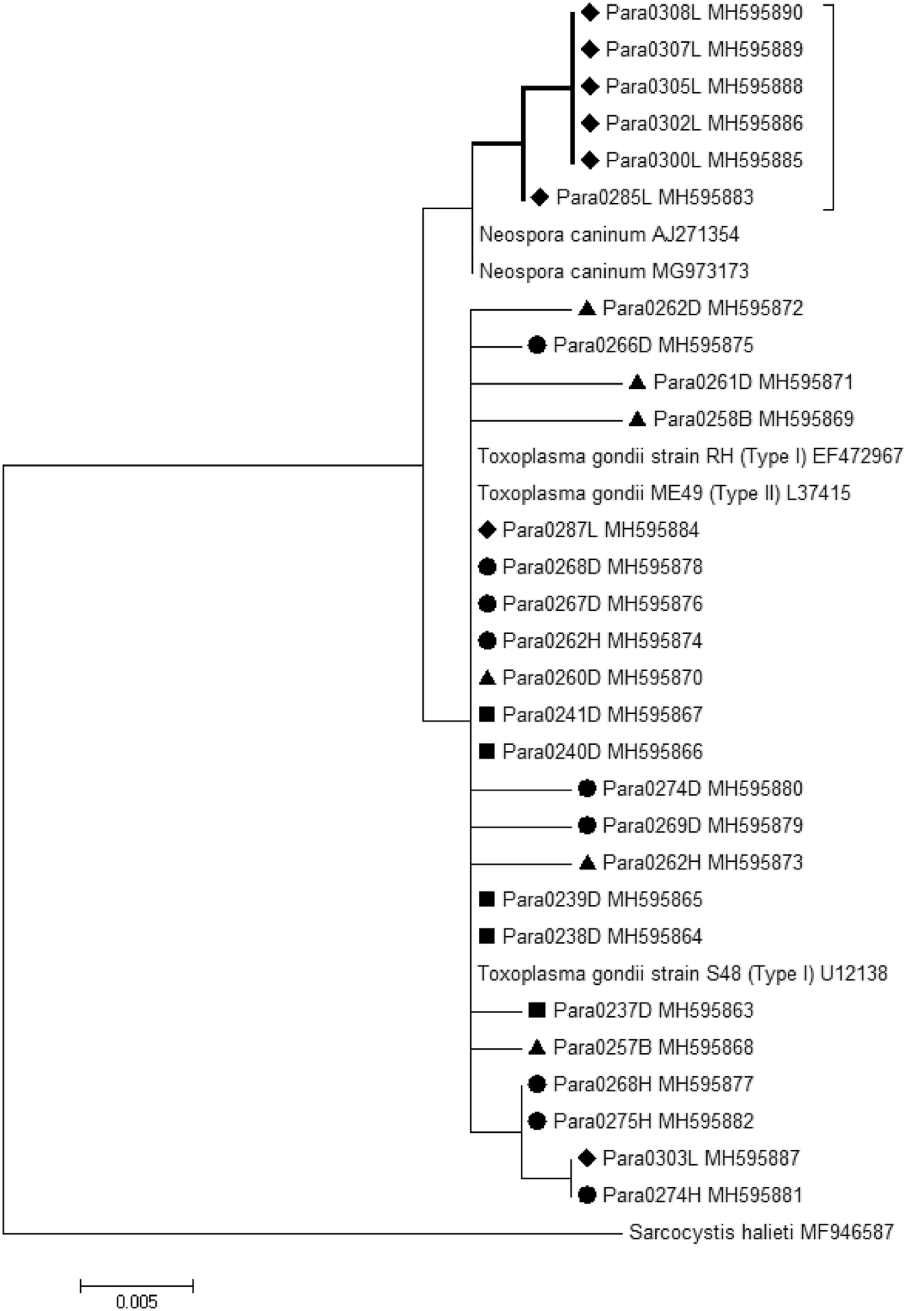
Phylogenetic analysis of the 18S gene of *Toxoplasma gondii* and *Neospora caninum-*like parasites in seals from northern and eastern Canada. No distinctive phylogenetic pattern was observed between seal species and *T. gondii* infections. A cluster of six ringed seals (bold lines) was found to be most closely related to *N. caninum*. Symbols represent seal type: ◆ ringed seal, ● harp seal, ■ grey seal, ▲ hooded seal. Organisms shown with no symbol are GenBank 18S sequences with their respective accession numbers indicated. The letter following the ParaXXXX sample number represents the tissue of infection: D, diaphragm; B, brain; H, heart muscle; L, lung.

A phylogenetic tree was made for the 18S gene region of *T. gondii* and the *N. caninum*-like parasites. Five of the six *N. caninum*-like positive tissue samples were 100% identical by sequencing (Fig. 2), whereas the sixth shared 99.8% identity. For *T. gondii*, some animals harbored single nucleotide polymorphisms (SNPs) of the parasite (hooded seal Para0262H *vs* hooded seal Para0262D: 99.1% identity; harp seal Para0274H *vs* harp seal Para0274D: 99.1% identity) (Fig. 2). There was insufficient information to determine whether SNPs were due to infection with more than one *T. gondii* strain, or because 18S is a multi-copy gene that may contain SNPs in one or more of its copies.

### 3.5 *Sarcocystis* sp. in seals belong to a unique genotype

**Fig. 3:**
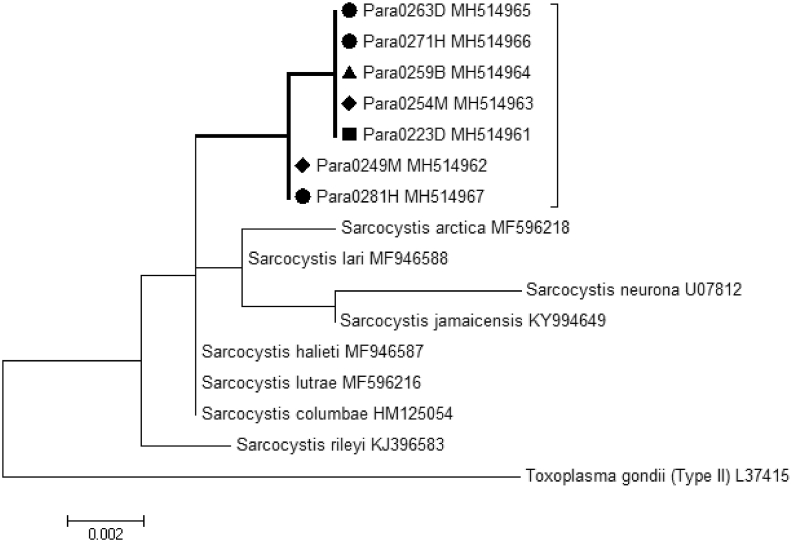
Phylogenetic analysis of 18S gene of *Sarcocystis* sp. in seals from northern and eastern Canada. All seven seal samples were grouped in a cluster (bold lines) that was distinct from other known *Sarcocystis* species. Symbols represent seal type: ◆ ringed seal, ● harp seal, ■ grey seal, ▲ hooded seal. Organisms shown with no symbol are GenBank 18S sequences with their respective accession numbers indicated. The letter following the ParaXXXX sample number represents the tissue of infection: D, diaphragm; B, brain; H, heart muscle; M, skeletal muscle.

Five of the seven *Sarcocystis* sp.-positive tissues had a single nucleotide polymorphism (SNP) that was distinct from known *Sarcocystis* spp. reference sequences archived in GenBank (Fig. 3). Ringed seal Para0249M and harp seal Para0281D had four SNPs, of which only two were identical to the SNPs of the other five seal tissues. Moreover, ringed seal Para0249M had two nucleotide variants in two of the SNPs. While *Sarcocystis* spp. are haploid in their intermediate hosts, 18S is a multi-copy gene and allelic variations in *Sarcocystis* spp. have been described previously (Aleman et al., 2016).

## 4 Discussion

Seal meat and organ tissues, including muscle, blubber, heart, liver, intestine, bones, cartilage, etc. (Pelly, 2001) have been consumed in Canada for thousands of years by indigenous people. They are generally eaten raw, rare, or undercooked depending on cultural habits, increasing the risk of parasites being transmitted to the consumer. Data from the present study suggests that Canadian seal meat and organ tissues may be a source of infection of *T. gondii.* As there is considerably less known about the infectivity and pathogenicity of *Sarcocystis* and *Neospora* in humans, the presence of these parasites in seals represents a lesser known risk to consumers.

In this study, we report the presence of DNA of Sarcocystidae parasites in all seal species tested. While DNA analysis may be less sensitive compared to serology, PCR and subsequent sequencing eliminates false-positive serological results due to cross-reaction between different species of the Sarcocystidae family (Gondim et al., 2017). Furthermore, serological tests should be carefully interpreted, depending on the antibodies used for testing, as some antibodies can be transmitted from mother to fetus (Montoya, 2002; Praet et al., 2010).

*Toxoplasma gondii* was reported in numerous wild otariid and phocid pinnipeds worldwide, including harbour seals, ringed seals, bearded seals, grey seals, hooded seals, and spotted seals (*Phoca largha*), but not ribbon seals (*Histriophoca fasciata*), in Canada and Alaska, USA (Lambourn et al., 2001; Dubey et al., 2003; Measures et al., 2004; Aguirre et al., 2007; Fujii et al., 2007; Simon et al., 2011; Rengifo-Herrera et al., 2012; Donahoe et al., 2014; Al-Adhami et al., 2016). Measures et al. (2004) reported seroprevalence of *T. gondii* in harbour, grey, and hooded seals, but not in harp seals, on the east coast of Canada. Oksanen and coworkers did not detect *T. gondii* in harp, ringed, and hooded seals from the Northeastern Atlantic using serology (Oksanen et al., 1998). Thus, we report harp seals as a new host for *T. gondii*.

Furthermore, we report evidence of vertical transmission (transplacental or transmammary) in one 4-day-old male harp seal pup (Para0262H). Vertical transmission may also have occurred in five *T. gondii* infected YOY ringed seals in our study but, as these animals were harvested by Inuit in September and eastern Arctic ringed seals are born mid-March to mid-April with two months of lactation, it is possible that they may have acquired infections via their diet which is initially pelagic crustaceans and later fish such as Arctic cod (*Boreogadus saida*) (see Smith, 1973). Muscle tissue of Arctic char (*Salvelinus alpinus*) and Atlantic salmon (*Salmo salar*) have recently been identified as *T. gondii* DNA-positive (Reiling and Dixon, 2019). Measures et al. (2004) reported one 10-day-old harbour seal pup and one 14-day-old grey seal pup seropositive to *T. gondii*, but they attributed seropositivity to maternal antibodies. Vertical transmission of *T. gondii* and *S. neurona* was documented in an aborted sea otter (*Enhydra lutra*) pup (Shapiro et al., 2016). Furthermore, co-infections with *T. gondii* and *S. neurona* have been reported (Gibson et al., 2011; Shapiro et al., 2016). Transmission of such protozoans to marine mammals, including cetaceans, in the marine environment is not fully understood and vertical transmission (exogenous or endogenous) may be one way to infect conspecifics or offspring (Worth et al., 2013; Donahoe et al., 2015; Iqbal et al., 2018).

Extralimital reports of ringed, grey, harp and hooded seals in southern waters such as southern Nova Scotia and New England (Lucas and McAlpine, 2002; Mignucci-Giannoni and Haddow, 2002), or even as far south as the Caribbean in the case of juvenile hooded seals (Mignucci-Giannoni and Odell, 2001), often involve seals that are sick and stranded. These seals may be taken into rehabilitation facilities, where they may be at greater risk of exposure to protozoans such as *N. caninum*, *Sarcocystis* spp. and *T. gondii* and where infected wild and domestic canids and felids contaminate coastal environments (Measures, 2004). For example, canids such as coyotes (*Canis latrans*) are known to venture onto ice in coastal environments to scavenge and predate seals (Way and Horton, 2004; Chubbs and Phillips, 2005). Canids, including coyotes, foxes (*Vulpes vulpes*) and wolves (*C. lupus*) are infected with *Sarcocystis* spp. and *N. caninum* (Dubey and Odening, 2001; Donahoe et al., 2015) but the relationship of these coccidians in canids with those in seals is unknown.

*Sarcocystis* spp. is reported in otariids [Guadalupe fur seals (*Arctocephalus townsendi*), northern fur seals (*Callorhinus ursinus*), and California sea lions (*Zalophus californianus*)] and phocids [harbour seals, northern elephant seals (*Mirounga angustirostris*) and Hawaiian monk seals (*Monachus schauinslandi*)] (Miller et al., 2001; Mylniczenko et al., 2008; Barbosa et al., 2015; Barbieri et al., 2016). *Sarcocystis neurona* DNA was detected in beach-cast otariids, phocids, sea otters and cetaceans in Oregon and Washington, USA and British Columbia, Canada (Gibson et al., 2011; Barbosa et al., 2015). *Sarcocystis* spp. has not been described in seals from Arctic Canada or eastern Canada. Thus, we report ringed seals, hooded seals, harp seals, and grey seals as new host reports in Canada.

*Neospora caninum* or *N. caninum*-like or “Coccidia C″ were reported in otariids (California sea lions, Guadalupe fur seals) and phocids (ringed seals, bearded seals, harbour seals, ribbon seals, Kuril harbour seals (*P.v. stejnegeri*), and spotted seals (Dubey et al., 2003; Fujii et al., 2007; Gibson et al., 2011). Because the genetic differences between *N. caninum* and *Hammondia heydorni* and their relationship to *T. gondii* have not been fully resolved (Mugridge et al., 1999; Mehlhorn and Heydorn, 2000; Dubey et al., 2002b), we consider *N. caninum* indistinguishable from *H. heydorni*. Furthermore, we consider *H. hammondii* indistinguishable from *T. gondii* in this study. In Alaska, seroprevalence of *N. caninum* was reported in harbour seals and ringed seals but not in bearded seals, spotted seals, or ribbon seals (Dubey et al., 2003). Our results provide new records of *N. caninum*-like parasites in Canadian ringed seals. It is not clear whether this suggests acute or systemic neosporosis as no histopathology was conducted on any of our samples. Furthermore, the 18S gene is conserved in some *Neospora* spp. and further sequencing will be needed to accurately identify the *N. caninum*-like parasites as *N. caninum* (Marsh et al., 1998). As noted above for *T. gondii*, *N. caninum-*like parasites were found in YOY ringed seals but we could not confirm transplacental infection for either parasite due to the date of collection.

Stranded marine mammals are often sick and do not represent the health of wild populations, thus prevalence of parasites and associated disease in carcasses or sick stranded animals, as frequently reported in the literature, may over-estimate the role of these parasites in wild populations. As *S. neurona* and other *Sarcocystis* spp. infections are associated with myositis, severe meningoencephalitis, and hepatitis in stranded marine mammals, the prevalence of *Sarcocystis* spp. in the wild population may be lower. In our study, apparently healthy seals were shot by Inuit hunters or under scientific permit and prevalence of protozoan infections may not be comparable to stranded animals. While some researchers described a novel *S. neurona* genotype in seals (Barbosa et al., 2015), we were unable to identify the closest relative to the genotype that was found in our study because many *Sarcocystis* spp. and *Neospora* spp. are identical in the 18S gene region that was analysed.

Our data also show that it is imperative to confirm PCR-positive results with sequencing. Because *Toxoplasma*, *Sarcocystis* and *Neospora* are very closely related to one another, it is possible to amplify more than one parasite species of the Sarcocystidae family with the same primers. Alternatively, more specific primers may be designed to eliminate false-positive PCR results, as was done in the present study using *Toxoplasma* B1 and 18S *Sarcocystis*-specific primers.

## 5 Conclusions

The results of this study demonstrate that DNA from parasites of the Sarcocystidae family, particularly *T. gondii* and *Sarcocystis* sp., is prevalent in tissues of northern and eastern Canadian seals. Although based only on the detection of parasite DNA, these findings nevertheless suggest that consumption of raw or undercooked seal meat or organ tissues can pose a risk of infection to consumers. For consumer safety, seal meat and other organ tissues should be thoroughly cooked or frozen. For example, freezing at −10 °C or lower for at least three days was shown to be sufficient for killing *T. gondii* and *Sarcocystis* spp. (Fayer, 1975; Srivastava et al., 1986; Kotula et al., 1991; El-Nawawi et al., 2008). The same protocols should be followed when feeding seal meat to dogs to prevent transmission and propagation of *N. caninum* or *N. caninum*-like parasites.

The limitations of this study are primarily due to sample size. With four different species of seals, varying in age, diet, behaviour and distribution, collected from five different harvest areas, an analysis of observed differences in prevalence of infection for each of three protozoan parasites is not possible. Moreover, there may be different modes of transmission or different exposure rates to the parasites because some species of seals (harp and hooded) undertake seasonal migrations to more southern waters. The types and numbers of tissues were also limited for some seals. Consequently, it is difficult to fully assess the risk to consumers except to state that these three parasites are present in Canadian pinnipeds and that further data are required to evaluate zoonotic risk.
